# Model simulations capture seasonal Arctic Haze and clean-air cycle better than satellite and reanalysis

**DOI:** 10.1038/s41598-025-29188-8

**Published:** 2025-12-01

**Authors:** Basudev Swain, Marco Vountas, Aishwarya Singh, Rui Song, Upasana Panda, Heiko Schellhorn, Linus Andrae, Adrien Deroubaix, Luca Lelli, Ankit Tandon, Akshaya Nikumbh, Sachin S. Gunthe

**Affiliations:** 1 Present Address: Department of Physics, Atmospheric, Oceanic and Planetary Physics, University of Oxford, Oxford, UK; 2 Institute of Environmental Physics, University of Bremen, Bremen, Germany; 3 Aerosol Chemistry Department, Max-Planck Institute for Chemistry, Mainz, Germany; 4 Kalinga Institute of Industrial Technology (KIIT) Deemed to be University, Bhubaneswar, India; 5 Max-Planck-Institut für Meteorologie, Hamburg, Germany; 6 Remote Sensing Technology Institute, German Aerospace Centre (DLR), Wessling, Germany; 7 Department of Environmental Sciences, Central University of Jammu, Jammu, India; 8 Department of Climate Studies, Indian Institute of Technology Bombay, Maharashtra, India; 9 Department of Civil Engineering, Indian Institute of Technology Madras, Chennai, India

**Keywords:** Climate sciences, Climate change, Climate and Earth system modelling

## Abstract

The Arctic is heating far more rapidly than the global mean, and clarifying the influence of aerosols in this intensification demands accurate and reliable observational records. The Arctic exhibits a distinct seasonal aerosol cycle, springtime ”Arctic Haze” with elevated AOD and summertime “Clean Air” with low AOD. Thus, it is critical to evaluate how well various datasets capture this seasonality relative to ground-based observations. This study analyzes spring and summer AOD variability using CAMSRA and MERRA-2 reanalyses, MODIS Terra and Aqua satellite observations, AERONET measurements, AEROSNOW retrievals, and GEOS-Chem model simulations. Results show that satellite-derived and satellite-assimilated reanalyses are far from capturing the expected seasonal Arctic Haze and Clean Air pattern, except at Bonanza Creek and Yakutsk, where anthropogenic pollution alters it. The inability of reanalyses to capture Arctic aerosol seasonality likely stems from the assimilation of satellite retrievals influenced by cloud contamination and surface reflection from snow and ice, as well as inherent biases in the underlying models used to generate these datasets. In contrast, AERONET observations and GEOS-Chem simulations consistently capture Arctic Haze in spring, driven by long-range transport, and Clean Air in summer, associated with efficient wet removal of aerosols. CAMSRA further underestimates emissions from Arctic forest fires and inadequately represents long-range pollution transport. These findings suggest that independent model simulations align more closely with ground-based observations than satellite products or reanalyses, and that adjusting wet-scavenging parameters to fit such reanalyses may misrepresent aerosol processes and their contribution to Arctic warming. Incorporating advanced retrieval algorithms like AEROSNOW into reanalyses offers a pathway to reduce these biases and improve representation of Arctic aerosol seasonality.

## Introduction

Arctic Amplification (AA) is a significant aspect of global climate change, as the Arctic is warming more rapidly than other parts of the globe^1^. Although the primary cause of Arctic temperature rise is due to the rising anthropogenic GHG levels^2^, aerosols also have an impact on the climate by influencing cloud characteristics and radiative forcing through their functions as ice nucleating particles (INP) and cloud condensation nuclei (CCN)^3^. Aerosol concentration in the Arctic peaks in spring (March to May) called Arctic Haze^4–6^, due to the influence of long-range transport from mid-latitudes showing significant seasonal variations. In contrast, during summer (June to August), the influx of transported aerosols decreases, natural aerosol sources become more dominant, and wet scavenging through precipitation leads to a reduced overall aerosol loading called clean air conditions^4,5,7,8^. These seasonal variations affect radiative balance, atmospheric circulation, and Arctic cloud characteristics^9,10^, However, due to a lack of observational data, the exact contribution of seasonal high (in spring) and low (in summer) aerosol load to Arctic Amplification is still not sufficiently understood^6,9,11–13^.

Field campaign initiatives that have yielded useful in situ aerosol measurements^14–17^, have provided valuable datasets, but their spatial and temporal coverage remains insufficient to characterize aerosol behavior across the central Arctic fully. Satellite-based observations have the potential to bridge this gap, offering large-scale estimates of aerosol optical depth (AOD). However, retrievals over the Arctic are highly uncertain due to challenges associated with high surface reflectance from snow and ice, as well as persistent cloud cover^6,7,18,19^. These limitations hinder accurate aerosol quantification in the Arctic, particularly over vast ice-covered regions, where direct observations remain sparse^19^. Using satellite-derived top-of-atmosphere reflectance data, different retrieval techniques have been devised to avoid these obstacles^20–23^. Nevertheless, the majority of such kind research are carried out on specific geographic areas, such as the Svalbard archipelago^20,21^, but the large portions of the Arctic are underrepresented.

Due to the scarcity of direct aerosol observations, reanalysis datasets^19,24,25^ and atmospheric models have been widely used to investigate Arctic aerosol seasonality^4,6,8,25,26^. Among these, the Copernicus Atmosphere Monitoring Service Re-Analysis (CAMSRA)^27,28^ provided by the European Centre for Medium-Range Weather Forecasts (ECMWF) and the Modern-Era Retrospective Analysis for Research and Applications, version 2 (MERRA-2)^29^ by NASA, are commonly used as surrogate observational datasets, particularly in regions where ground-based measurements are limited or nonexistent. However, these reanalysis products are highly dependent on satellite data assimilation^27–29^, which can introduce biases due to issues such as surface reflectivity over ice-covered regions and persistent cloud contamination. Consequently, these datasets may not fully capture Arctic aerosol seasonality, leading to discrepancies compared to independent observations^19,24^. Further, it is important to note that although satellite AOD observations are only assimilated up to $70^{\circ }$N, the assimilation process propagates satellite-like patterns into regions north of this latitude. This can be attributed to several indirect mechanisms: (a) model dynamics and physics that spread the influence of assimilated data into adjacent regions; (b) atmospheric transport processes such as advection, diffusion, and mixing that move aerosols poleward; (c) background error covariance within the assimilation system, which extends observational influence beyond the direct assimilation domain; and (d) long-range transport during spring, which enables assimilation-driven adjustments in the lower Arctic to extend into the high Arctic^28,30–32^.

To address the need for improved aerosol retrievals in Arctic cryospheric regions, the AEROSNOW algorithm^12^ has been developed, offering enhanced AOD retrievals specifically designed for snow- and ice-covered surfaces. By incorporating refined parameterizations of the bidirectional reflectance distribution function (BRDF) on the surface^33^ and advanced cloud screening techniques^34^, AEROSNOW deliver an accurate representation of AOD variability in the Arctic, covering the central cryosphere from 2003 to 2011^12^.

Thus limited measured coverage in the Arctic has led many studies to use reanalysis products as a baseline^4,6,8,19,25,26^. These datasets, however, depend on satellite assimilation, which can be biased by persistent clouds and the reflective nature of snow- and ice-covered areas. It is therefore essential to assess whether reanalysis and satellite estimates can accurately capture seasonal Arctic Haze and clean air conditions, alongside model simulations, when compared with ground-based AERONET observations and AEROSNOW retrievals.

In this study, we investigate Arctic aerosol seasonality using multiple datasets, including the CAMSRA^27,28^ and MERRA-2^29^, AERONET ground-based measurements^35^, AEROSNOW satellite retrievals^12^, Moderate Resolution Imaging Spectroradiometer (MODIS) Terra and Aqua observations^36^, and GEOS-Chem chemical transport model simulations^37^. Our analysis shows that model simulations captures seasonal Arctic Haze and clean air conditions more accurately than both satellite and reanalysis datasets. These findings provide a valuable foundation for ongoing and future research that relies on satellite and reanalysis products to study Arctic aerosol seasonality and its implications for Arctic warming.

## Results

### Comparison of AODs from all datasets with ground-based measurements

**Fig. 1:**
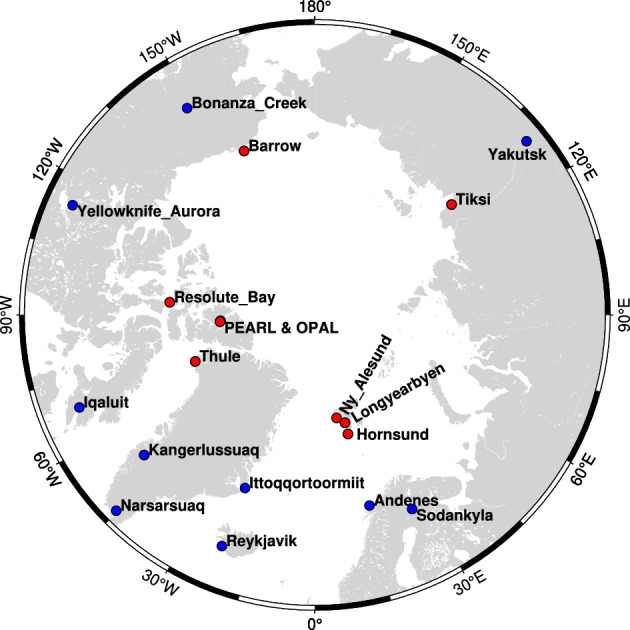
Location of eighteen different AERONET sites used in this study. The low-Arctic ($60^{\circ }$N to $70^{\circ }$N) and high-Arctic ($70^{\circ }$ to $80^{\circ }$N) AERONET stations are shown as blue and red dots, respectively.

**Fig. 2:**
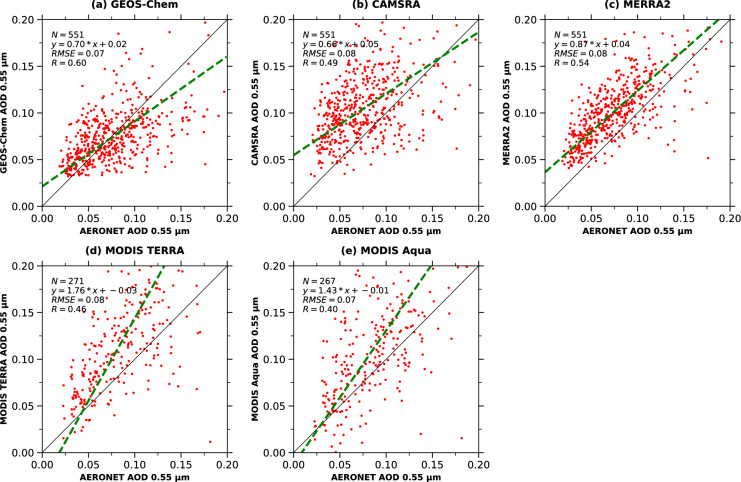
Evaluation of AOD obtained from all the platforms with respect to ground-based AERONET measurements. (**a**, **b**, **c**, and **d**) shows the evaluation of monthly mean AOD between GEOS-Chem model, CAMSRA, MERRA-2 reanalysis, MODIS Terra, and MODIS Aqua, respectively.

In the next step, AODs from all different datsets, such as reanalysis, satellite retrievals, and model estimations are evaluated with the AOD measured at eighteen different ground stations (Fig. 1) from 2003 to 2011. Fig. 2(a-e) shows the evaluation between GEOS-Chem 3D chemical transport model, CAMSRA and MERRA-2 reanalysis data, MODIS Terra and Aqua satellite observations with the AOD measured at ground-based AERONET stations, respectively. We use the reduced major axis (RMA) fitting technique^38,39^) for the data analysis since the AOD observations on both platforms are susceptible to measurement errors^21,40^. This approach ensures a more stable and dependable association between the datasets and is especially appropriate in situations where both variables in the regression (AOD values from each platform) are prone to measurement errors.

There are other ways to minimize the errors, such as minimizing the triangle or the perpendicular distance, and we decided to use the RMA, which involves reducing the triangle. Other researchers, such as^39,41–44^, have employed RMA in their study of air pollution and climate sciences. This method is particularly suitable for Arctic applications because it performs well under conditions of low signal-to-noise ratios, sparse observational data, and extreme seasonal variability.

By combining all these eighteen AERONET stations, the coefficient of correlation (Pearson correlation coefficient, R) is obtained as 0.60, 0.49, 0.54, 0.46, and 0.40 for GEOS-Chem model simulations, CAMSRA, MERRA-2, MODIS Terra, and Aqua satellite observations, respectively (Fig. 2a-e). Given the challenge of these results over the Arctic, we consider R values of $>0.5$ being adequate^12^. From the evaluation, it is clear that the model AOD simulations are having better agreement with the ground-based observations than the CAMSRA, MERRA-2 reanalysis, and MODIS observations.

### Spring and summertime seasonal Arctic AOD

The seasonality of Arctic AOD during spring and summer is obtained from satellite observations, ground-based measurements, reanalyses assimilated data, and model-simulated AOD over low-Arctic and high-Arctic ground-based measurement sites. We then present the seasonal cycle of AOD derived from each datasets to show that model simulation is following the ground-based observations, while limited (quality-assured) Arctic AOD satellite retrievals assimilated reanalysis data is reversing the Arctic AOD observed seasonality during Arctic spring and summer seasons.

#### Satellite and reanalysis are far from capturing Arctic aerosol seasonality at low- and high-Arctic AERONET sites

It is important to assess the seasonal variability of AOD from all the platforms as the variations of reflection from snow regions, large solar zenith angles (SZAs), long-range transport air pollutants, persistent cloud presence, and intensity of precipitation changes from the low-latitude Arctic (60N-70N) to high Arctic (70N-90N) during spring to summer season transition. The low- and high-Arctic AERONET locations used in this study are marked as blue and red dots in Fig. 1 respectively.

**Fig. 3:**
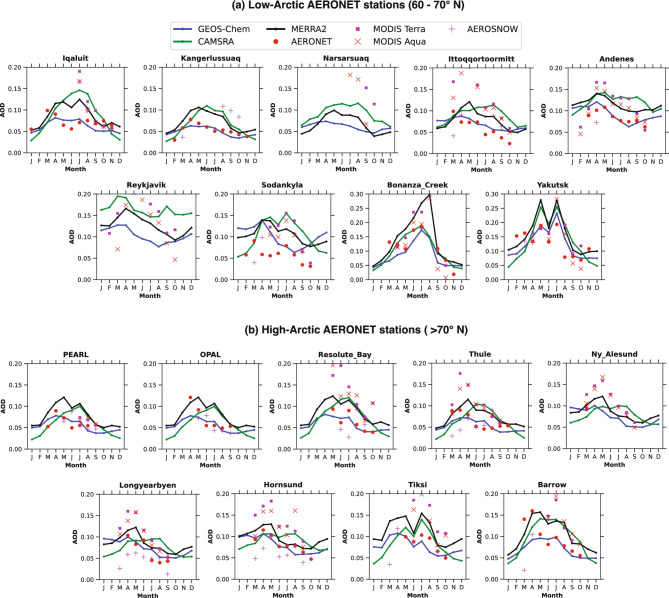
Seasonal variability of AODs from all the platforms over low- and high-Arctic AERONET stations. a, and b shows the seasonal variability of the GEOS-Chem model, CAMSRA, MERRA-2 reanalysis, MODIS Terra, and MODIS Aqua AOD over low- and high-Arctic regions, respectively.

Fig. 3a presents the seasonal variability of AOD across nine low-Arctic AERONET sites. The GEOS-Chem model shows good agreement with ground-based AERONET observations at low-Arctic stations such as Iqaluit, Kangerlussuaq, Ittoqqortoormitt, Andenes. However over Sodankylä, the GEOS-Chem model is overestimating AOD during spring. In contrast, CAMSRA, which assimilates only satellite observations, closely tracks space-borne AOD estimates. MERRA-2, which assimilates both satellite and AERONET measurements, shows slightly improved performance over CAMSRA but still largely mirrors the satellite observations (Fig. 3a). At locations without AERONET stations, such as Narsarsuaq and Reykjavik, both the reanalysis products continue to track satellite-derived AOD, whereas the GEOS-Chem model simulations differ substantially. Exceptions include Bonanza Creek and Yakutsk, near the Arctic Circle ($60^{\circ }$ N), where all datasets show similar seasonality, with high AOD (0.2) in summer, reflecting the influence of continental anthropogenic pollution from Canada and China, which alters the typical Arctic aerosol pattern. AOD values exceeding 0.2 in Siberia during summer are linked to biomass burning^19,45^. Overall, except for Bonanza Creek and Yakutsk, CAMSRA reanalysis shows higher AOD in summer, reflecting its satellite assimilation (MODIS Terra and Aqua, and AATSR), while GEOS-Chem simulations capture the typical Arctic seasonal pattern of higher AOD in spring and lower AOD in summer, consistent with ground-based AERONET observations.

Further, Fig. 3b shows the seasonal variability of AOD across nine high-Arctic AERONET sites. In these sites, CAMSRA, and MERRA2 reanalysis data mirrors satellite observations at most of these stations, showing higher AOD in summer and lower AOD in spring, apart from Ny-Alesund, Longyearbyen, and Hornsund, where all the datasets in both of these seasons show a bit closer seasonality. Conversely, ground-based AERONET measurements and GEOS-Chem simulations depict the opposite, with higher AOD in spring (in line with Arctic Haze events) and lower AOD in summer. While we have not explicitly quantified the influence of frontal uplift linked to the North Atlantic storm track at each station, the observed station-to-station differences in AOD seasonality are consistent with local variability in aerosol sources, retrieval challenges over reflective snow- and ice-covered surfaces, and meteorological effects such as wet deposition and precipitation, which can further modulate AOD^5,26,46^. Notably, both low- and high-Arctic regions show a opposite AOD seasonality in CAMSRA, MERRA-2, and satellite data compared to GEOS-Chem and AERONET observations (Fig. 3a,b). This discrepancy could be attributed to the assimilation of low-quality satellite AOD to create reanalysis datasets, which changes the seasonal pattern, particularly in spring and summer. While satellite data offers broad spatial coverage for the globe, it often lacks accuracy in regions with reflective surfaces, such as snow and ice, or in areas with frequent cloud cover^12^. These challenges are especially pronounced in the Arctic, complicating the retrieval of reliable AOD from space-based instruments.

During the spring season, the Arctic experiences a high influx of aerosols transported from lower latitudes, primarily due to the long-range transport of pollutants from Europe, Asia, and North America^5^. In theory, this should result in a clear peak in AOD during spring in reanalysis and satellite data, which is captured by GEOS-Chem model simulations and ground-based AERONET observations (Fig. 3a,b). However, this could be attributed to low-quality satellite observations and its further assimilation into reanalysis datasets, they tend to underrepresent this aerosol loading. The bright surface of snow and ice often confounds satellite retrieval algorithms, leading to an overestimation of AOD in the low and high Arctic regions (Fig. 3a,b). Consequently, the assimilation of these cloud contaminated satellite data into reanalysis products could be suppressing the expected springtime AOD peak, resulting in a less pronounced aerosol seasonality in the reanalysis datasets compared to GEOS-Chem model outputs.

**Fig. 4:**
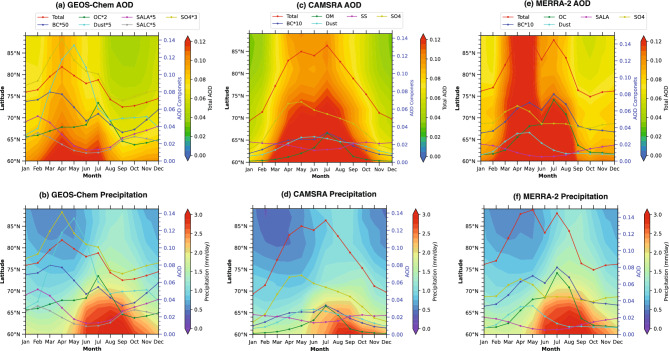
The Zonally and monthly averaged Aerosol Optical Depth and precipitation contour plots with different aerosol components from the GEOS-Chem model and CAMSRA, MERRA-2 reanalysis from 2003 to 2011. (**a**), and (**b**) present contour plots for Aerosol Optical Depth and precipitation from GEOS-Chem. (**c**), and (**d**) present contour plots for Aerosol Optical Depth and precipitation from CAMSRA. (**e**), and (**f**) present contour plots for Aerosol Optical Depth and precipitation from MERRA-2. Seasonal AOD components shown for GEOS-Chem, the Individual components are multiplied by scaling factors to highlight seasonal variations. Thus, the yellow and green lines may appear higher than total AOD.

The impact of low-quality satellite observations are even more evident during the summer season. In summer, natural aerosol sources such as sea salt, biogenic emissions, and dust become more prominent in the Arctic^4,5^, while anthropogenic aerosol transport diminishes. However, the satellite and satellite observation assimilated reanalysis datasets often fail to capture these low aerosol loads accurately due to the same challenges of bright surface reflectance and cloud interference (Fig. 3a,b). Furthermore, the zonal average seasonal plots (Fig. 4a–f) show that the model simulation captures low AOD during summer, coinciding with higher precipitation (Fig. 4a,b). In contrast, CAMSRA and MERRA-2 display high summer AOD (JJA) despite the higher precipitation, likely reflecting an underestimation of wet scavenging in the reanalyses. MERRA-2 shows a slight reduction in AOD during summer, partially aligning with the precipitation pattern. This is further discussed in details in coming subsection.

Overall, both satellite observations and satellite-assimilated reanalysis products estimate Arctic aerosol seasonality opposite to observations, particularly during the spring-to-summer transition, whereas the model closely aligns with ground-based measurements, capturing high AOD in spring and low AOD in summer across both low- and high-Arctic regions. This opposite AOD seasonal variability estimated by satellite and reanalysis products can obscure important seasonal dynamics, such as the shift from anthropogenic to natural aerosol sources and the role of precipitation in removing aerosols from the atmosphere. As a result, studies that rely on these satellite and reanalysis datasets may miss key aspects of aerosol-precipitation interactions and their effects on Arctic Amplification, leading to an incomplete understanding of the region’s aerosol-climate feedback^13^.

### Zonal-averaged AOD seasonality in the Arctic: Links to seasonal precipitation patterns

The Arctic experiences high precipitation during summer, which reduces aerosol load through wet-deposition processes, as highlighted in previous studies^5,9,26^. To examine the apparent mismatch between the elevated summer AOD estimated by reanalysis products, opposite to observations, and the seasonal precipitation patterns, we analyzed zonally averaged seasonal AOD alongside zonally averaged seasonal precipitation in Fig. 4a–f. To better understand these relationships, Fig. 4(a-f) illustrates spring and summertime total aerosol optical depth-averaged zonally and corresponding precipitation, plotted with aerosol types from the GEOS-Chem model and CAMSRA, MERRA-2 datsets. This visualization allows for a deeper assessment of the co-variation between aerosols and precipitation.

The Arctic region between $60^{\circ }$N and $90^{\circ }$N exhibits elevated aerosol loads in spring, a period of relatively low precipitation (Fig. 4b,d,f). In contrast, GEOS-Chem simulations show a reduction in aerosol levels during summer, coinciding with higher precipitation (Fig. 4a,b), reflecting effective wet-removal processes. In the reanalysis datasets, however, summer AOD remains high in CAMSRA and only slightly reduced in MERRA-2 despite increased precipitation (Fig. 4c–f), indicating weak coupling between AOD and precipitation in reanalysis products. GEOS-Chem produces higher spring AOD and lower summer AOD (Fig. 4a), consistent with seasonal precipitation and wet-removal processes^47^. The model also accounts for seasonal shifts in atmospheric circulation that reduce long-range pollutant transport during summer^37^. In contrast, CAMSRA and MERRA-2 show minimal co-variation between AOD and precipitation (Fig. 4b,d), with elevated summer AOD coinciding with high precipitation. Consequently, reanalysis products overestimate summer AOD and fail to capture the observed Arctic aerosol seasonality seen in GEOS-Chem simulations. Although MERRA-2 and GEOS-Chem use the same meteorological fields as input, subtle differences in AOD patterns still arise. These differences can be attributed to factors such as spatial resolution, deposition parameterizations, representation of detailed aerosol chemistry, and transport schemes^37^. GEOS-Chem explicitly simulates wet removal and long-range transport processes, capturing the seasonal Arctic Haze in spring and Clean Air conditions in summer, whereas MERRA-2, as a reanalysis product, may smooth or redistribute aerosols due to coarser resolution and assimilation of satellite observations. This highlights that even with identical meteorology, differences in model design and process representation are critical for accurately reproducing Arctic AOD patterns.

In the Arctic, particularly between $70^{\circ }$ and $90^{\circ }$N, discrepancies between reanalysis datasets and model simulations are especially pronounced (Fig. 4a–f). Reanalysis datasets show little co-variation between AOD and precipitation, likely due to both the underrepresentation of wet-scavenging in the models underlying CAMSRA and MERRA-2 and the assimilation of satellite products. As noted by^48^, satellite observations often fail to capture wet-scavenging events, so aerosols removed by precipitation are not reflected in the retrievals, resulting in overestimated AOD. In addition, satellite-derived AOD products are subject to several limitations that can lead to overestimation under cloudy or precipitating conditions. First, wet scavenging of aerosols by precipitation is not directly observed by satellites, so retrievals may not reflect the actual reduction of aerosol loading in the atmosphere^48,49^. Second, aerosols located beneath cloud layers are often not sampled, resulting in incomplete vertical coverage of the aerosol column. Third, hygroscopic growth under high relative humidity enhances particle size and optical properties, thereby increasing the retrieved AOD even when the actual dry aerosol mass remains low^50^.

While precipitation patterns (Fig. 4b,d,f) are similar across GEOS-Chem, CAMSRA, and MERRA-2 between $70--90^{\circ }$N, the observed differences in AOD suggest that aerosol removal processes, particularly dry deposition, play a significant role. Studies have shown that dry deposition is a crucial mechanism for removing aerosols in the Arctic, especially during spring when Arctic Haze events are prevalent. For instance^51^, highlighted the importance of dry deposition in the Arctic, noting that deposition fluxes are influenced by both dry and wet removal processes, as well as model precipitation and aerosol aging. Additionally^52^, found that large variations exist in the total black carbon deposition in the Arctic, emphasizing the need for accurate representation of deposition processes in models. These findings underscore the necessity of incorporating both wet and dry removal processes to accurately simulate Arctic aerosol seasonality and to understand the discrepancies observed between models and reanalyses.

**Fig. 5:**
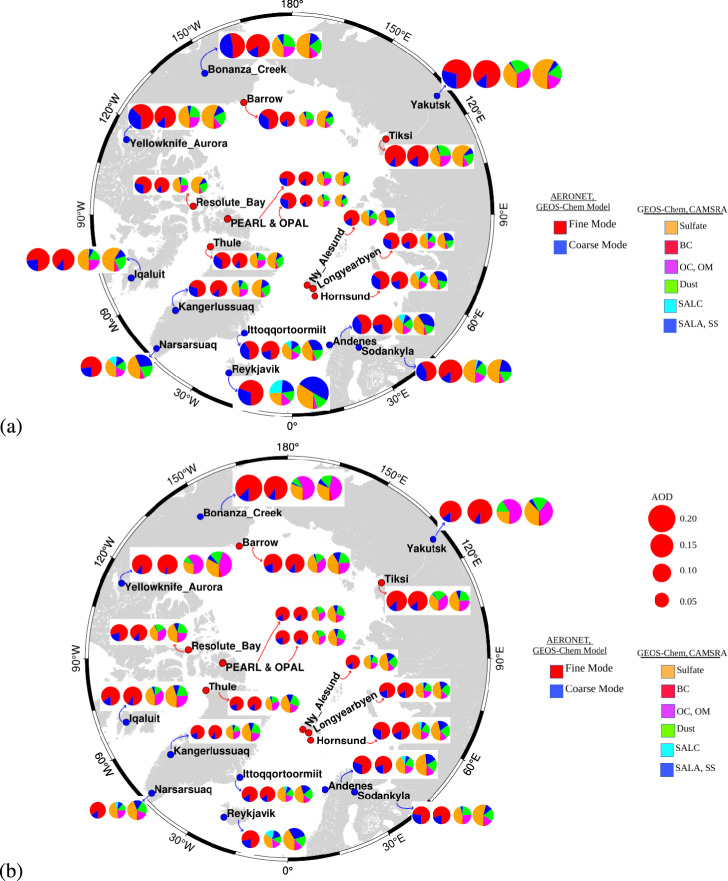
Arctic polar map for Spring and Summertime average of AOD speciations from GEOS-Chem model simulations and CAMSRA reanalysis data over low- and high-Arctic AERONET sites from 2003 to 2011. (**a**), and (**b**) shows the spring and summer seasonal average of GEOS-Chem model, CAMSRA reanalysis AOD speciations respectively. The red-blue colored pie-charts presented in left-most sides shows the fine and coarse model AOD obtained from AERONET (first pie-chart at left side) and model simulations (second pie-chart at left side) respectively, whereas the colored pie-charts shows the AOD speciations obtained from model simulations (third pie-chart) and CAMSRA (fourth pie-chart) reanalysis respectively.

Finally, cloud contamination in retrieval algorithms, particularly over snow- and ice-covered surfaces, further contributes to retrieval uncertainties. These factors together explain why satellite instruments, including MODIS and AATSR, tend to overestimate AOD in the presence of precipitating or humid cloud conditions. Further, when the high-AOD satellite observations are assimilated, reanalysis products inherit this bias. Consequently, the elevated summer AOD in reanalysis datasets, despite high precipitation, likely reflects both satellite assimilation together with limited wet-scavenging representation in the underlying models. On the other hand, GEOS-Chem model simulations generally predict lower AOD values in the high Arctic (between $70^{\circ }$ and $90^{\circ }$N) during the summer months (Fig. 4a), driven by the contribution of natural aerosol sources such as sea salt and biogenic emissions, which are more prevalent during this period (Fig. 5a,b). Moreover, GEOS-Chem model simulations tend to offer a more detailed representation of aerosol processes in the high Arctic, including the influence of localized sources and the efficiency of wet scavenging processes in this region^4,37^. This results in lower modeled AOD values compared to the reanalysis datasets, which often fail to capture these nuances due to the challenges of satellite data assimilation in the region.

Overall, the zonal and seasonal comparison underscores the limitations of reanalysis datasets in capturing the co-variation between zonally averaged AOD and precipitation. In contrast, the GEOS-Chem model, which simulates aerosol-precipitation nexus in greater detail^4^, provides a better representation of seasonal AOD variation with precipitation in the Arctic. Satellite observations and satellite-assimilated reanalysis products, however, deviate substantially from observed seasonal aerosol–precipitation patterns.

#### Seasonal AOD component variations over low- and high-Arctic regions

Speciated AOD shows greater variability than total AOD between CAMSRA reanalysis and GEOS-Chem model simulations over the low Arctic ($60^{\circ }$N to $70^{\circ }$N) and the high Arctic ($70^{\circ }$N to $90^{\circ }$N), revealing distinct seasonal differences (Fig. 5a,b). We compared only CAMSRA speciated AOD with GEOS-Chem simulations because GEOS-Chem is well-parameterized and validated against observations in prior studies^4,6^. Furthermore, CAMSRA speciated AOD is considered in our analysis, as both CAMSRA and MERRA-2 reanalyses exhibit similar speciation patterns^24^.

In the low Arctic, which experiences more direct anthropogenic influence from mid-latitudes during spring, CAMSRA underestimates OC and dust by approximately 70% and 4%, while overestimating BC, SS, and $\hbox {SO}_4$ by 85%, 64%, and 19%, respectively (Fig. 5a). For instance, at Barrow (Alaska), previous studies^4,5^ have reported that GEOS-Chem accurately captures the springtime Arctic haze characterized by elevated BC and OC, while CAMSRA underestimates OC, consistent with our findings. Similarly, at Ny-Ålesund (Svalbard), comparisons by^4,11^ indicated that reanalysis datasets fail to fully represent OC contributions from long-range transport of biomass burning aerosols from Europe and Asia, corroborating our observation of a 78% underestimation of OC in the high Arctic.

During summer, lower-Arctic regions, including stations such as Zeppelin, are more affected by regional biomass burning^7^, as long-range transport is reduced due to atmospheric circulation patterns. CAMSRA underestimates OC by 35% and overestimates BC by 150% compared to GEOS-Chem simulations (Fig. 5b). This aligns with findings by^53,54^ who reported that regional boreal forest fires in northern Russia and Scandinavia substantially contribute to summer aerosol loading in the lower Arctic, which is systematically lower in reanalyses than in model simulations.

In the high Arctic CAMSRA depicts as smaller value of OC by 41% and overestimates BC by 120% in summer (Fig. 5b) than model estimations. These discrepancies indicate an underrepresentation of the transport of boreal fire smoke into the high-Arctic sea ice region, which is consistent with observational studies by^55,56^ showing that carbonaceous aerosol transport from mid-latitudes to the high Arctic is episodic but significant during fire events. Notably, the OC contribution to total AOD and to the total carbonaceous fraction (BC+OC) remains higher than BC at these stations, emphasizing the underestimation of OC-driven climate impacts by CAMSRA reanalysis.

Across both spring and summer, the long-distance transport of pollutants from agricultural and industrial burning in Europe, North America, and Asian regions, as well as regional Arctic biomass burning, are underestimated in CAMSRA relative to GEOS-Chem (Fig. 5a,b). This underestimation is particularly evident when comparing station-level observations, at Barrow, spring OC peaks exceed those in CAMSRA by up to 70%, while over low-and high-Arctic OC peaks during summertime fire episodes are underestimated by 40%.

In the higher Arctic, CAMSRA depicts a higher value of SS by 315% (Fig. 5b), consistent with previous studies^57^ suggesting that coarse-mode particles are poorly represented in reanalysis datasets due to limitations in parameterizing local sea-salt emissions and transport. Discrepancies in fine-mode and coarse-mode AOD are also evident, GEOS-Chem overestimates fine-mode AOD during spring haze events at Ny-Ålesund and Barrow, while summer observations show comparable fine- and coarse-mode values between GEOS-Chem and AERONET.

Fig. 5(a,b) demonstrates that across both seasons, dust and sea salt make only a minor contribution to the total AOD in the Arctic, whereas biomass burning aerosols dominate. In summary, CAMSRA reanalysis depicts a higher value of OC from both long-range transport and regional biomass burning at both low- and high-Arctic stations during spring and summer, highlighting the need for improved representation of carbonaceous aerosols in Arctic reanalyses.

### The spatial distribution of aerosol seasonality in the central Arctic sea-ice region

The central Arctic sea ice region is devoid of high-resolution spatial AOD datasets from the ground as well as from the satellite. However, the recently developed AEROSNOW algorithm fills this data gap by retrieving high-resolution spatial AOD datasets from the Advanced Along-Track Scanning Radiometer (AATSR) satellite. Further, AATSR’s dual-view capability reduces surface reflection effects over snow and ice, but uncertainties remain due to residual cloud contamination, calibration drift, challenges in surface reflectance characterization, low solar angles, and limited spatial–temporal coverage, all of which can affect Arctic AOD retrieval accuracy. Thus, the AEROSNOW datasets^12^ provide a unique opportunity to assess the impact of the assimilation of low-quality assured MODIS datasets on the seasonal AOD distribution of the reanalysis data as well as the GEOS-Chem model simulations.

**Fig. 6:**
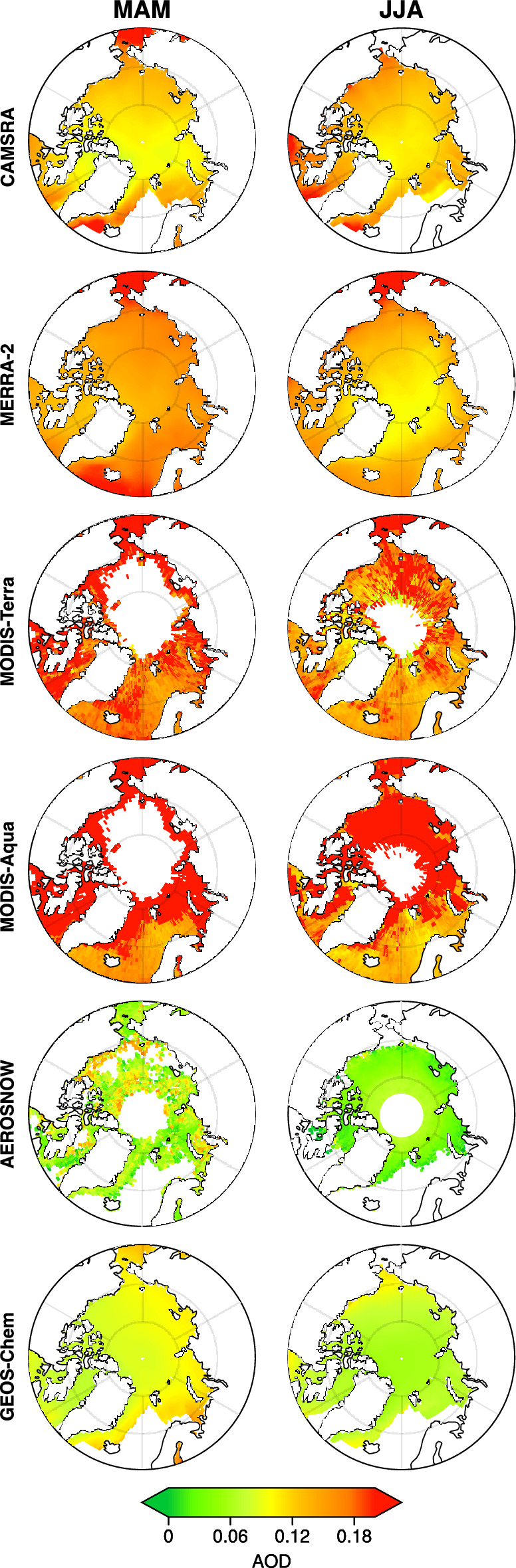
Spring and summertime spatial distribution of AODs from CAMSRA, MERRA-2 MODIS, AEROSNOW, and GEOS-Chem model over the central Arctic sea-ice region. Mean seasonal AOD during spring (left side) and summer (right side) from CAMSRA, MERRA-2, MODIS, AERONSOW, and GEOS-Chem model in central Arctic covered with sea-ice. These AODs are averaged from 2003 to 2009.

In the CAMSRA and MERRA-2 reanalysis dataset, the inherent limitations of satellite-based AOD retrievals from MODIS and AATSR, particularly over highly reflective surfaces like sea ice, lead to inaccurate spatial patterns that do not fully represent the true aerosol distribution in this region. Satellites struggle to distinguish between aerosols and the bright background of snow and ice, as well as to penetrate the prevalent cloud cover in the central Arctic, leading to an overestimation of AOD (Fig. 6). This issue is compounded by the fact that satellite algorithms are not optimized for the unique environmental conditions of the central Arctic.

During spring, long-range transported anthropogenic aerosols from mid-latitude regions are expected to dominate, creating a higher aerosol load over the central Arctic, called Arctic Haze events. However, the reanalysis datasets, influenced by the assimilation of low-quality satellite data, often over-represent this aerosol influx. The misrepresentation results in a more uniform and artificially high AOD spatial distribution over the sea-ice region (Fig. 6), which fails to capture the real variations associated with aerosol transport patterns. This inaccurate spatial mapping obscures important features like Arctic haze events that occur during spring with intense aerosol-cloud interactions, ultimately limiting the understanding of how aerosols affect Arctic climate processes during spring in CAMSRA, and MERRA-2 reanalysis data (Fig. 6).

In summer, the limitations of satellite-based reanalysis datasets become even more pronounced. As natural aerosols, such as sea salt and biogenic particles, become more prominent and anthropogenic influences diminish, the overall AOD in the central Arctic is expected to be lower. However, wet removal processes, which remove aerosols through precipitation, further reduce the aerosol load. Reanalysis datasets, however, tend to overestimate AOD in the central Arctic (Fig. 6). The low-quality satellite data assimilation often masks the spatial variability associated with these processes, leading to a flatter, less realistic representation of AOD over the central Arctic sea-ice region in reanalysis datasets. The impact of highly reflective sea ice coverage over the central Arctic on the low-quality MODIS satellite observations is further visible spatially during both spring and summer seasons in Fig. 6 as the lower AOD values are observed over dark open ocean Areas of northern Europe while extremely high values are over the sea ice region as well as over the transitional areas between the open dark ocean and sea ice regions (Fig. 6, MODIS Terra and Aqua). This is primarily because the algorithms used in both MODIS Terra and Aqua are mainly for dark targets rather than highly reflective snow and ice regions^12^.

In contrast, GEOS-Chem model-only simulations and advanced retrieval algorithms like AEROSNOW provide a more accurate representation of the spatial distribution of AOD over the central Arctic (Fig. 6). GEOS-Chem model simulations, which do not rely on satellite data, and well parameterized with respect to the Arctic conditions, offer a more detailed depiction of spatial distribution of AOD over central Arctic sea-ice^4^. These GEOS-Chem model simulations take into account the complex dynamics of wet scavenging, long-range transport, and regional within the Arctic aerosol sources, allowing for more precise spatial variability in AOD^4^. For example, models can simulate higher AOD in areas experiencing long-range aerosol transport and lower AOD where wet scavenging is most effective during summer. At Barrow observations show the classic Arctic Haze pattern–maxima in late winter–spring and minima in summer and models capture the timing reasonably well but often miss the magnitude and sometimes the summer minimum. Long-term in-situ records at this site show higher spring scattering and sulfate (haze) and low summer loading, confirming the canonical cycle^58^. This nuanced spatial pattern is often missed by reanalysis datasets that rely heavily on the assimilation low quality flagged MODIS satellite data.

Whereas, the AEROSNOW, a satellite-based retrieval scheme specifically designed for the Arctic, also gives significant improvement in aerosol optical depth spatial distribution in the central Arctic sea-ice region (Fig. 6). Unlike standard satellite algorithms, AEROSNOW is optimized to handle the challenges of snow and ice reflectivity and incorporates more stringent surface parameterizations and cloud screening techniques. This results in a more accurate depiction of spatial distribution of AOD, particularly in areas where traditional satellite retrievals fail. AEROSNOW captures finer spatial details of aerosol distribution, allowing for a better understanding of how aerosols interact with the polar environment in both the seasons (MAM and JJA).

**Fig. 7:**
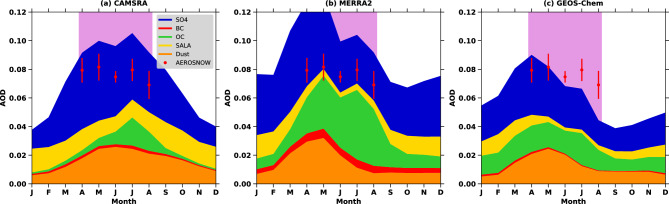
Seasonally averaged stack plot of various AOD speciations over central Arctic sea-ice regions from CAMSRA, MERRA-2 and GEOS-Chem model simulation. The season-wise fluctuations in aerosol optical depth in the central Arctic sea-ice zone, using AEROSNOW-derived data by using the mean of nine years (2003-2011) in black colour. The mean AOD values are represented by black points, while the vertical lines indicate one standard deviation for AOD retrievals from AEROSNOW. Panels (**a**), (**b**), AND (**c**) display stacked plots illustrating the different aerosol species, as simulated by CAMSRA, MERRA-2, and GEOS-Chem respectively.

It is worth noting that the assimilation of low-quality satellite AOD measurements to create reanalysis datasets leads to inaccurate spatial distributions of AODs in the central Arctic cryospheric zones, with significant over-representation of AOD variability. Whereas, GEOS-Chem model-only simulations and AEROSNOW retrievals provide a clearer and more accurate spatial picture, capturing the complexities of aerosol transport, removal, as well as seasonal changes in Arctic^5,6^. This is further confirmed in the seasonal average of aerosol component stack for the central Arctic sea-ice region of CAMSRA, MERRA-2, and GEOS-Chem model with respect to AEROSNOW presented in Fig. 7(a,b,c), wherein the reanalysis products, it is clearly visible that there is less impact of precipitation as these reanalysis dataset shows higher aerosol load during summer, while the GEOS-Chem model (Fig. 7c) is in line with the AEROSNOW observations, with little underestimations during summer. According to^4,10,59^, the model may have not been fully capturing the natural emissions from oceanic regions free of snow and ice, which could account for this summertime underestimation. Additionally^60^, describes how higher iodine emissions may contribute to the frequent creation of new particles above the high Arctic pack ice, as well as higher wet-scavenging in models due to higher level of precipitation. Furthermore, even though the stringent cloud masking algorithm has reduced the impact of cloud cover, it may still have an effect on AEROSNOW retrieval due to cloud contamination^34^. It is not entirely possible to rule out the potential influence of residual cloud affects for the AOD retrieval, despite the implementation of strict cloud masking^6,34^. These more refined approaches are essential for improving our understanding of aerosol impacts on the Arctic climate system.

**Fig. 8:**
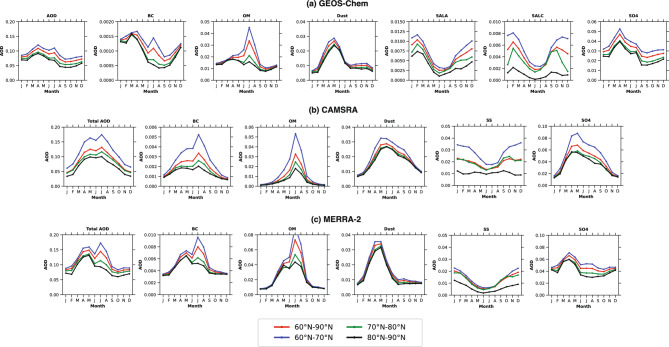
Seasonal variability of total AOD and its speciations over various latitude belts over the Arctic from GEOS-Chem model simulations, CAMSRA, and MERRA-2 reanalysis data from 2003-2011. The seasonal variations in total and speciated AODs across the Arctic ($60--90^{\circ }$N) from 2003 to 2019 are analyzed using GEOS-Chem model outputs, CAMSRA, and MERRA-2 aerosol reanalysis.

This typical feature of GEOS-Chem model simulated, CAMSRA and MERRA-2 AOD is further propagating latitude-wise variations (Presented in Fig. 8a-c). The seasonal average of AOD components across different Arctic latitudinal regions indicates a decrease in AOD from the lower to the higher Arctic regions. The model simulations show higher total AOD ($60--70^{\circ }$N) during spring, whereas the CAMSRA reanalysis exhibits the opposite trend compared to the model simulations (Fig. 8a, b). In contrast, MERRA-2 is somewhat closer to the GEOS-Chem model simulations (Fig. 8a, c) than CAMSRA. Further, the total AOD for the $80--90^{\circ }$N high latitude belt increased minutely from spring to summer in reanalysis data. This indicates that the overall AOD exhibits a diminishing gradient with increasing latitude is characterized by a larger amplitude in summer than in spring in the reanalysis data compared to GEOS-Chem model simulations. This is probably because the reanalysis data shows less aerosol wet removal during summertime travel from source regions to the high Arctic. Furthermore, in comparison to the simulations produced by the GEOS-Chem model, the CAMSRA and MERRA-2 latitudinal AOD gradient is higher (Fig. 8a-c). In comparison to the reanalysis datasets, this indicates that the GEOS-Chem model in the Arctic removes aerosols more effectively.

## Discussion

Assessing Arctic aerosol seasonality using GEOS-Chem, CAMSRA, and MERRA-2 involves multiple sources of uncertainty. Although GEOS-Chem, CAMSRA, and MERRA2 rely largely on similar emission inventories (see Table S1), differences in their aerosol estimates and seasonal variability are primarily driven by model configurations and data assimilation techniques rather than the emissions themselves. Further, GEOS-Chem, as a chemical transport model, depends on prescribed emissions inventories, meteorological fields, and chemical mechanisms. While it explicitly represents aerosol transport, chemistry, and deposition, uncertainties in emissions, reaction rates, and boundary conditions can affect simulated aerosol optical depth (AOD), especially in the central Arctic, where observational constraints are sparse. Multi-model evaluations^7,61^ indicate that GEOS-Chem generally reproduces the seasonal cycle and mean aerosol distributions but often overestimates spring AOD and underestimates summer AOD. These biases likely arise from simplified aerosol process representations, such as secondary organic aerosol formation, long-range transport, and wet/dry deposition, as well as regional variability and interannual changes. It is worth noting that the GEOS-Chem simulations were performed at $2^{\circ } \times 2.5^{\circ }$ resolution, whereas CAMSRA and MERRA-2 have finer resolutions of $0.75^{\circ } \times 0.75^{\circ }$ and $0.5^{\circ } \times 0.625^{\circ }$, respectively. Despite its coarser resolution, GEOS-Chem reproduces AOD at high-Arctic sites more accurately than the reanalyses. This is likely due to its explicit treatment of aerosol processes, including long-range transport, wet deposition, and detailed chemistry, which are critical for capturing Arctic aerosol seasonality. In contrast, reanalyses, though finer in resolution, rely heavily on assimilated satellite AOD, which can introduce biases over reflective snow- and ice-covered surfaces and under frequent cloud cover. These results highlight that spatial resolution alone does not guarantee improved model–observation agreement; accurate representation of aerosol processes and removal mechanisms is equally important^62^.

CAMSRA and MERRA-2 are reanalysis systems that assimilate satellite observations to produce consistent aerosol fields. While data assimilation can correct model biases, it inherits uncertainties from satellite retrievals, which struggle over highly reflective surfaces and in cloud- or precipitation-affected regions. These retrievals can miss wet-scavenging events, leading to overestimated summer AOD despite high precipitation that should reduce aerosol concentrations^48^. The central Arctic sea-ice region remains particularly data-sparse, limiting confidence in seasonal variability estimates. Differences in model physics, chemistry, and assimilation strategies further contribute to divergent seasonal cycles among datasets, even when using similar emissions inputs.

A recent study by^24^ analyzed Arctic AOD trends and biomass burning events but did not address several issues tackled here. They compared reanalysis products (e.g., CAMSRA, MERRA-2) with the same satellite datasets used in their assimilation, leading to artificially close agreement. Their analysis excluded the central Arctic sea-ice region, where coverage before AEROSNOW was sparse, and did not compare seasonal AOD variations against AERONET at individual sites, relying only on pan-Arctic averages. Summer (JJA) AOD from AERONET was generally lower than reanalysis estimates–except at Bonanza Creek, influenced by Alaskan forest fires–suggesting possible overestimation. No independent model simulations were used to evaluate seasonal variability, and their focus was on long-term trends rather than reanalysis accuracy in representing Arctic aerosol seasonality.

Our study addresses these gaps by integrating satellite retrievals, reanalysis products, AERONET observations, and GEOS-Chem simulations. Direct comparison of reanalysis data with AERONET measurements reveals a reversed seasonal cycle in CAMSRA and MERRA-2–higher AOD in summer and lower in spring–opposite to the typical Arctic pattern of elevated spring AOD from long-range transport and reduced summer AOD from wet scavenging. In contrast, GEOS-Chem tends to underestimate summer AOD over the central Arctic, likely due to missing natural aerosol sources and loss processes.

These findings demonstrate that evaluating reanalysis datasets without independent validation can mask systematic biases. Integrating multiple datasets and interpreting reanalysis-based results with caution is essential for accurately characterizing Arctic aerosol seasonality and its climatic impacts.

## Conclusion

The seasonal distribution of AOD in the Arctic indicates that satellite-derived reanalysis datasets such as CAMSRA and MERRA-2, which rely on low-quality AOD retrievals, poorly capture seasonal Arctic Haze in spring and clean air conditions in summer. These reanalysis datasets tend to show higher AOD in summer and lower AOD in spring, contrary to ground-based AERONET measurements and GEOS-Chem model simulations, which correctly reflect high AOD in spring due to long-range aerosol transport and summer AOD reductions from precipitation. The discrepancies are particularly pronounced in the middle and high Arctic sea-ice regions, where retrieval challenges over highly reflective surfaces and cloud cover limit satellite accuracy. Moreover, the lack of seasonal and zonal co-variation between AOD and precipitation in these datasets further contributes to these errors.

Analysis of aerosol composition reveals that CAMSRA underestimates organic carbon (OC) and overestimates black carbon (BC) across both low- and high-Arctic regions during spring and summer compared to GEOS-Chem simulations. In spring, CAMSRA underestimates OC by 70% in the low Arctic and 78% in the high Arctic, while overestimating BC by 85% and 35%, respectively. These discrepancies indicate inadequate representation of long-range transport of anthropogenic carbonaceous aerosols from mid-latitude regions including Asia, Europe, and North America. During summer, CAMSRA underestimates OC by 35% in the low Arctic and 41% in the high Arctic, while overestimating BC by 150% and 120%, respectively. OC contributes 25% of total AOD in spring and 40% in summer, while BC contributes only 1.5% in spring and 1% in summer. The persistent underestimation of OC and overestimation of BC highlights limitations in capturing regional biomass burning impacts on Arctic aerosols and underscores the need for improved portrayal of these constituents in reanalysis datasets. Accurate modeling of OC and BC is essential for assessing the effects of long-range transport and increasing regional biomass burning on the rapidly changing Arctic environment.

Furthermore, this study suggests that Arctic aerosol research relying on satellite and reanalysis datasets as a observational baseline should avoid adjusting wet-scavenging parameters merely to align model AOD with reanalysis values. Such adjustments risk producing unrealistic results, misrepresenting the seasonal aerosol-Arctic warming relationship, and generating misleading conclusions. Prioritizing realistic aerosol processes over fitting models to potentially biased datasets is critical.

These outcomes underscore the importance of assimilating advanced retrieval algorithms, such as AEROSNOW, to develop improved Arctic reanalysis datasets. Integrating AEROSNOW data can refine model simulations, reducing biases present in current datasets like CAMSRA, and enhance our understanding of the seasonal behavior of Arctic aerosols and their implications for climate processes, including Arctic amplification.

## Datasets and method

### Model simulations

We utilized version 12.2.1 of the GEOS-Chem global 3D model, available at http://acmg.seas.harvard.edu/geos/^37^. The Modern Era Retrospective Reanalysis2 (MERRA-2) is a high-resolution, six-hourly assimilated meteorological dataset developed by NASA’s Goddard Modeling and Assimilation Office (GMAO) and is integrated into this model to enhance its accuracy. MERRA-2 provides comprehensive atmospheric data, combining observations from various datasets and advanced modeling techniques to represent global meteorological conditions. The model utilizes this dataset alongside detailed representations of gas-aerosol interactions, aerosol processes, and the complex chemistry between ozone (O3), nitrogen oxides (NOx), and hydrocarbons (HCs). Through fully coupled simulations, the model captures the intricate relationships between these components, allowing for more precise simulations of atmospheric dynamics and pollutant interactions. This approach is critical for understanding air quality, climate processes, and chemical interactions in the atmosphere, as discussed in previous studies^63,65,66^.

The choice of the GEOS-Chem model for this study is primarily motivated by the desire to take advantage of the MERRA-2 meteorological dataset. This dataset is particularly very much suitable for modeling the Arctic region meteorology, as highlighted by^4^. GEOS-Chem offers better compatibility with the Arctic-specific processes than the Coupled Model Inter-comparison Project (CMIP6) models^66^. CMIP6 models do not integrate updated emission inventories, which are essential for an accurate representation of regional pollution sources^67^. Additionally, these models face challenges in simulating important processes, such as the long-range transport and deposition of aerosols, which are very important to the Arctic conditions. Moreover, CMIP6 models lack detailed vertical distribution data, a crucial factor for effectively modeling aerosol transport within the region. As highlighted by^67^, these limitations make GEOS-Chem a more appropriate choice for our study, ensuring a more accurate representation of the complex atmospheric conditions in the Arctic.

The model incorporates carbonaceous aerosols, such as primary organic carbon (POC) and black carbon (BC), in accordance with normal GEOS-Chem protocols^68^. The other fractions are classified as hydrophilic, while it is believed that 50% of POC emissions and 80% of BC emissions are hydrophobic. Hydrophobic aerosols change into hydrophilic forms over a 1.15-day aging period^69^, increasing their vulnerability to moist deposition.

In order to simulate aerosol dynamics, the model also incorporates a number of aerosol processing schemes, namely^70^ for sea salt^70,72,73^for aerosol optical parameters^73^, for dust mobilization, wet deposition^47^, and dry deposition^74^.

A 10-minute transit time step and a 20-minute chemical and emissions time step were employed for our simulations. The model has 72 vertical levels that reach up to 0.01 hPa and runs at $2^{\circ } \times 2.5^{\circ }$ horizontal resolution, or 220 km $\times$ 50 km in the high Arctic latitudes of the OPAL region^37,75^. The $2^{\circ } \times 2.5^{\circ }$ simulations’ boundary conditions were taken from earlier global simulations with a $4^{\circ } \times 5^{\circ }$ resolution. The first three years (1999-2002) are referred to as a spin-up period in order to allow for model stabilization over the 13-year simulation period, which runs from 1999 to 2011.

The GEOS-Chem simulated fine ($\tau _\text {f,GEOS-Chem}$) and coarse ($\tau _\text {c,GEOS-Chem}$) mode components of AOD consisting of mineral dust, fine and coarse mode sea salt (SALA), sulfate, BC, and OC in the fine mode^76^. The following procedure is used to calculate the fine and coarse mode AOD:

1 $$\begin{aligned} \begin{aligned}&\tau _{\text {f}} = \sum _{\text {l}=1}^{72} (\tau _\text {f,l,SO4} + \tau _\text {f,l,BC} + \tau _\text {f,l,OC} + \tau _\text {f,l,SALA} + \tau _\text {f,l,dust} ),\\&\tau _{\text {c}} = \sum _{\text {l}=1}^{72} (\tau _\text {c,l,SALA} + \tau _\text {c,l,dust} ), \end{aligned} \end{aligned}$$

where l represents the 72 vertical levels.

To compute the total AOD at 550 nm, the Global Aerosol Data Set (GADS) was used^71^^72^. GADS provides robust data on aerosol optical properties, size-ranges for different relative humidity (RH) values^77^. These data are processed using the Mie calculation^78^. Then the final AOD is obtained as follows:

2 $$\begin{aligned} \tau = \frac{3}{4} \frac{Q_\text {ext} M}{r_\text {eff} \rho } \end{aligned}$$

The extinction coefficient, denoted as $Q_\text {ext}$, is obtained from GADS, while $M$ represents the mass of a column. The aerosol mass density is denoted by $\rho$^79^. The various inventories for different emission sources considered in this study are presented in Table S1.

### Reanalysis datasets

The Copernicus Atmosphere Monitoring Service (CAMS) Reanalysis (CAMSRA) dataset^28^, for the years 2003–2011, was used for the reanalysis. Aerosol species like sulfate, dust, sea salt, organic matter, and black carbon are included in this model, which is powered by the Integrated Forecasting System (IFS) of ECMWF^28^. Observations and satellite-derived AOD at 550 nm from MODIS and AATSR are included in this dataset. This dataset provides better aerosol speciation than previous reanalyses (i.e. MACC).

The Modern-Era Retrospective Analysis for Research and Applications, Version 2 (MERRA-2)^29^ is NASA’s latest global atmospheric reanalysis produced by the Global Modeling and Assimilation Office (GMAO). Covering the satellite era from 1980 to the present, MERRA-2 integrates a wide range of satellite observations into the GEOS modeling framework to generate consistent, gridded atmospheric fields, including meteorology, trace gases, and aerosols. It is the first NASA reanalysis to assimilate space-based aerosol optical depth (AOD) retrievals, enabling improved representation of aerosol distributions and their interactions with radiation and clouds. While highly valuable for climate and atmospheric research, MERRA-2’s accuracy depends on the quality of assimilated satellite retrievals and model parameterizations, which can introduce regional and seasonal biases, particularly in data-sparse regions like the central Arctic.

There are a few reanalysis datasets such as MERRA-2^29^, MACC, CAMS, and CAMSRA, these reanalysis datasets are widely used globally as well as over the Arctic region. The main reasons for the selection of the CAMSRA and MEERA2 dataset for our studies are the following;

1. This CAMSRA AOD dataset is representative of two other reanalysis datasets such as MACC, and CAMS, as the CAMSRA datasets are created following the steps of MACC^28^.

2. The CAMSRA datasets were created by using MODIS as well as AATSR datasets during our study period^28^, and in our study, we have used AATSR data to retrieve AOD over the central Arctic cryosphere by developing and employing the AEROSNOW algorithm^12^. This further makes the assessment as well as makes easier to strengthen our hypothesis of our study over the central Arctic region.

3. All reanalysis data sets (such as MERRA-2, MACC, CAMS, and CAMSRA) show similar seasonality of the AOD as per^24^. Thus, considering MERRA-2 together with CAMSRA reanalysis data, which is representative of MACC, CAMS is the more suitable choice for our study.

### Satellite observations

**MODIS Terra and Aqua:** Collection 6.1 Dark and deep Blue target retrievals served as the foundation for the MODIS AOD data for Terra and Aqua^36^. The MODIS data at 550 nm are a level 3 product with a daily temporal resolution and a spatial resolution of $1^{\circ } \times 1^{\circ }$. The daily mean MODIS AOD is binned to the monthly mean for AOD climatology.

**AEROSNOW Retrieval:** The AEROSNOW Arctic aerosol retrieval process concentrated at the dynamic Arctic sea-ice zones, a zone that is currently undergoing rapid transformations. Due to various difficulties posed by reflection from snow-ice-covered zones and the presence of low-hanging clouds, which hinder the collection and observation of aerosol, an innovative retrieval strategy was implemented, as outlined in^12^. This method leveraged data from the AATSR instrument on the ENVISAT satellite. The algorithm, comprehensively described in^12^, is designed to detect and isolate cloud-free conditions at high latitudes and subsequently derive AOD.

The research focuses on two distinct Arctic periods: springtime (April and May) as well as summertime (June to August). The spring and summertime are marked by different aerosol sources–spring is very much impacted by the long-distance transport of anthropogenic particles, while summer is dominated by aerosol originating from local Arctic emissions. Spring is characterized by minimal precipitation, whereas summer sees significant rainfall, as noted in^5,9^. By applying the AEROSNOW retrieval during these times, the study establishes a reference to evaluate the accuracy of CMIP6 climate models for Arctic aerosol load. This comparison offers valuable information on how well the CMIP6 simulations perform against satellite data as well as its correlation at varying precipitation patterns.

### Ground-based AERONET observations

We incorporate the pertinent AERONET AOD along with the AEROSNOW AOD retrievals and GEOS-Chem estimations. This ground-located AOD measurement network provides observations of AOD at different wavelengths bands by taking sunphotometers instrument^80^ in seven different spectral channels, including 340, 380, 440, 500, 670, 870, and 1020 nm in each 15-minute^35^. The quality-assured AERONET version 3 level 2 data, available at https://aeronet.gsfc.nasa.gov/ used in this investigation. Fig. 1 displays the chosen high-arctic AERONET station locations. The fine and coarse mode aerosol observational information is obtained from AERONET at 500 nm^81,82^. The FM AOD at 550 nm was extrapolated using the FM spectral derivative at 500 nm, whereas the CM AOD at 500 nm was estimated to be equal to the 550 nm value^19^.

Likewise, wavelength conversion was necessary to compare the AERONET AOD observed at 500 nm with the GEOS-Chem AOD (modeled at 550 nm). For comparisons with GEOS-Chem, we calculated the AERONET at AOD at 550 nm using the Ångström exponent from AOD at 500 and 870 nm. For the GEOS-Chem AOD, the AERONET observations are then monthly averaged and is collocated at 25 km radius around the AERONET stations with the model simulations.

Furthermore, all datasets are considered for the period 2003–2011, as our AEROSNOW retrieval^12^ covers the same timeframe. This ensures a more appropriate assessment of other satellite-based measurements and their subsequent impact on Arctic aerosol seasonality in reanalysis datasets.

## Supplementary Information

Supplementary Information.

## Data Availability

All data supporting the conclusions of this paper are available either through the links provided below or upon request. AERONET Version 3 Level 2 data http://aeronet.gsfc.nasa.gov (last access: 10 July 2023; AERONET, 2022). MODIS data-assimilation-quality AOD at https://ladsweb.modaps.eosdis.nasa.gov/missions-and-measurements/products/MOD08_D3. CAMSRA AOD at https://www.ecmwf.int/en/research/climate-reanalysis/cams-reanalysis (last access: 10 July 2023). MERRA-2 data is available ate https://gmao.gsfc.nasa.gov/gmao-products/merra-2/data-access_merra-2/. AEROSNOW methodology is available at https://amt.copernicus.org/articles/17/359/2024/amt-17-359-2024.html. GEOS-Chem model data and source code is available at http://acmg.seas.harvard.edu/geos/.

## References

[1] Rantanen, M. et al. The arctic has warmed nearly four times faster than the globe since 1979. *Commun. Earth & Environ.* **3**, 168. 10.1038/s43247-022-00498-3 (2022).

[2] Gillett, N. P. et al. Attribution of polar warming to human influence. *Nat. Geosci.* **1**, 750–754 (2008).

[3] Rosenfeld, D. et al. Flood or drought: how do aerosols affect precipitation?. *science* **321**, 1309–1313 (2008). 18772428 10.1126/science.1160606

[4] Breider, T. J. et al. Multidecadal trends in aerosol radiative forcing over the arctic: Contribution of changes in anthropogenic aerosol to arctic warming since. *J. Geophys. Res. Atmospheres* **122**, 3573–3594. 10.1002/2016JD025321 (1980) https://agupubs.onlinelibrary.wiley.com/doi/pdf/10.1002/2016JD025321.

[5] Willis, M. D., Leaitch, W. R. & Abbatt, J. P. Processes controlling the composition and abundance of arctic aerosol. *Rev. Geophys.* **56**, 621–671 (2018).

[6] Swain, B. et al. Aerosols in the central arctic cryosphere: satellite and model integrated insights during arctic spring and summer. *Atmospheric Chem. Phys.* **24**, 5671–5693 (2024).

[7] Sand, M. et al. Aerosols at the poles: an aerocom phase ii multi-model evaluation. *Atmospheric Chem. Phys.* **17**, 12197–12218. 10.5194/acp-17-12197-2017 (2017).

[8] Sand, M. et al. Aerosol absorption in global models from aerocom phase iii. *Atmospheric Chem. Phys.* **21**, 15929–15947. 10.5194/acp-21-15929-2021 (2021).

[9] Schmale, J., Zieger, P. & Ekman, A. M. Aerosols in current and future arctic climate. *Nat. Clim. Chang.* **11**, 95–105. 10.1038/s41558-020-00969-5 (2021).

[10] Schmale, J. et al. Pan-arctic seasonal cycles and long-term trends of aerosol properties from ten observatories. *Atmospheric Chem. Phys. Discuss.* **2021**, 1–53 (2021).

[11] Breider, T. J. et al. Annual distributions and sources of arctic aerosol components, aerosol optical depth, and aerosol absorption. *J. Geophys. Res. Atmospheres* **119**, 4107–4124 (2014).

[12] Swain, B. et al. Retrieval of aerosol optical depth over the arctic cryosphere during spring and summer using satellite observations. *Atmospheric Meas. Tech. (AMT)* **17**, 359–375 (2024).

[13] Swain, B. et al. Insights of aerosol-precipitation nexus in the central arctic through cmip6 climate models. *npj Clim. Atmospheric Sci.* **8**, 103 (2025).

[14] Hoffmann, A. et al. Remote sensing and in-situ measurements of tropospheric aerosol, a pamarcmip case study. *Atmospheric Environ.* **52**, 56–66. 10.1016/j.atmosenv.2011.11.027 (2012).

[15] Wendisch, M. et al. The arctic cloud puzzle: Using acloud/pascal multiplatform observations to unravel the role of clouds and aerosol particles in arctic amplification. *Bull. Am. Meteorol. Soc.* **100**, 841–871. 10.1175/BAMS-D-18-0072.1 (2019).

[16] Ohata, S. et al. Arctic black carbon during pamarcmip 2018 and previous aircraft experiments in spring. *Atmospheric Chem. Phys.* **21**, 15861–15881. 10.5194/acp-21-15861-2021 (2021).

[17] Shupe, M. D. et al. Overview of the MOSAiC expedition: Atmosphere. *Elem. Sci. Anthropocene* **10**, 00060, 10.1525/elementa.2021.00060 (2022). https://online.ucpress.edu/elementa/article-pdf/10/1/00060/780058/elementa.2021.00060.pdf.

[18] Toth, T. D. et al. Minimum aerosol layer detection sensitivities and their subsequent impacts on aerosol optical thickness retrievals in calipso level 2 data products. *Atmospheric Meas. Tech.* **11**, 499–514. 10.5194/amt-11-499-2018 (2018). 10.5194/amt-11-499-2018 PMC8051137 33868502

[19] Xian, P. et al. Arctic spring and summertime aerosol optical depth baseline from long-term observations and model reanalyses - part 2: Statistics of extreme aod events, and implications for the impact of regional biomass burning processes. *Atmospheric Chem. Phys.* **22**, 9949–9967. 10.5194/acp-22-9949-2022 (2022).

[20] Istomina, L. G., von Hoyningen-Huene, W., Kokhanovsky, A. A., Schultz, E. & Burrows, J. P. Remote sensing of aerosols over snow using infrared aatsr observations. *Atmospheric Meas. Tech.* **4**, 1133–1145. 10.5194/amt-4-1133-2011 (2011).

[21] Mei, L. et al. Aerosol optical depth retrieval in the arctic region using modis data over snow. *Remote. Sens. Environ.* **128**, 234–245. 10.1016/j.rse.2012.10.009 (2013).

[22] Mei, L. et al. On the retrieval of aerosol optical depth over cryosphere using passive remote sensing. *Remote. Sens. Environ.* **241**, 111731. 10.1016/j.rse.2020.111731 (2020).

[23] Mei, L. et al. Retrieval of aerosol optical thickness in the arctic snow-covered regions using passive remote sensing: Impact of aerosol typing and surface reflection model. *IEEE Transactions on Geosci. Remote. Sens.* **58**, 5117–5131. 10.1109/TGRS.2020.2972339 (2020).

[24] Xian, P. et al. Arctic spring and summertime aerosol optical depth baseline from long-term observations and model reanalyses-part 1: Climatology and trend. *Atmospheric Chem. Phys.* **22**, 9915–9947 (2022).

[25] von Hardenberg, J. et al. Aerosol optical depth over the arctic: a comparison of echam-ham and tm5 with ground-based, satellite and reanalysis data. *Atmospheric Chem. Phys.* **12**, 6953–6967. 10.5194/acp-12-6953-2012 (2012).

[26] Ren, L. et al. Source attribution of arctic black carbon and sulfate aerosols and associated arctic surface warming during 1980–2018. *Atmospheric Chem. Phys.* **20**, 9067–9085. 10.5194/acp-20-9067-2020 (2020).

[27] Inness, A. et al. The macc reanalysis: an 8 yr data set of atmospheric composition. *Atmospheric chemistry physics* **13**, 4073–4109 (2013).

[28] Inness, A. et al. The cams reanalysis of atmospheric composition. *Atmospheric Chem. Phys.* **19**, 3515–3556 (2019).

[29] Gelaro, R. et al. The modern-era retrospective analysis for research and applications, version 2 (merra-2). *J. climate* **30**, 5419–5454. 10.1175/JCLI-D-16-0758.1 (2017). 10.1175/JCLI-D-16-0758.1 PMC6999672 32020988

[30] Benedetti, A. et al. Aerosol analysis and forecast in the european centre for medium-range weather forecasts integrated forecast system: 2. data assimilation. *J. Geophys. Res. Atmospheres* **114** (2009).

[31] System description and data assimilation evaluation. Randles, C. et al. The merra-2 aerosol reanalysis, 1980 onward. part i. *J. climate* **30**, 6823–6850 (2017). 10.1175/JCLI-D-16-0609.1 PMC5859955 29576684

[32] Lamarque, J.-F. et al. The atmospheric chemistry and climate model intercomparison project (accmip): overview and description of models, simulations and climate diagnostics. *Geosci. Model. Dev.* **6**, 179–206 (2013).

[33] Kokhanovsky, A. A. & Breon, F.-M. Validation of an analytical snow brdf model using parasol multi-angular and multispectral observations. *IEEE Geosci. Remote. Sens. Lett.* **9**, 928–932 (2012).

[34] Jafariserajehlou, S. et al. A cloud identification algorithm over the arctic for use with aatsr-slstr measurements. *Atmospheric Meas. Tech.* **12**, 1059–1076 (2019).

[35] Holben, B. N. et al. An emerging ground-based aerosol climatology: Aerosol optical depth from aeronet. *J. Geophys. Res. Atmospheres* **106**, 12067–12097, 10.1029/2001JD900014 (2001). https://agupubs.onlinelibrary.wiley.com/doi/pdf/10.1029/2001JD900014.

[36] Levy, R. et al. The collection 6 modis aerosol products over land and ocean. *Atmospheric Meas. Tech.* **6**, 2989–3034 (2013).

[37] Bey, I. et al. Global modeling of tropospheric chemistry with assimilated meteorology: Model description and evaluation. *J. Geophys. Res. Atmospheres* **106**, 23073–23095. 10.1029/2001JD000807 (2001).

[38] Hirsch, R. M. & Gilroy, E. J. Methods of fitting a straight line to data: Examples in water resources 1. *JAWRA Journal of the American Water Resources Association* **20**, 705–711 (1984).

[39] Ayers, G. Comment on regression analysis of air quality data. *Atmospheric Environ.* **35**, 2423–2425 (2001).

[40] Giles, D. M. et al. Advancements in the aerosol robotic network (aeronet) version 3 database-automated near-real-time quality control algorithm with improved cloud screening for sun photometer aerosol optical depth (aod) measurements. *Atmospheric Meas. Tech.* **12**, 169–209 (2019).

[41] Keene, W. C., Pszenny, A. A., Galloway, J. N. & Hawley, M. E. Sea-salt corrections and interpretation of constituent ratios in marine precipitation. *J. Geophys. Res. Atmospheres* **91**, 6647–6658 (1986).

[42] Arimoto, R. et al. Trace elements in the atmosphere over the north atlantic. *J. Geophys. Res. Atmospheres* **100**, 1199–1213 (1995).

[43] Freijer, J. I. & Bloemen, H. J. T. Modeling relationships between indoor and outdoor air quality. *J. Air & Waste Manag. Assoc.* **50**, 292–300 (2000). 10680359 10.1080/10473289.2000.10464007

[44] Wang, T. et al. Relationships of trace gases and aerosols and the emission characteristics at lin’an, a rural site in eastern china, during spring 2001. *J. Geophys. Res. Atmospheres* **109** (2004).

[45] McCarty, J. L. et al. Reviews and syntheses: Arctic fire regimes and emissions in the 21st century. *Biogeosciences* **18**, 5053–5083. 10.5194/bg-18-5053-2021 (2021).

[46] Sand, M., Berntsen, T. K., Seland, Ø. & Kristjánsson, J. E. Arctic surface temperature change to emissions of black carbon within arctic or midlatitudes. *J. Geophys. Res. Atmospheres* **118**, 7788–7798. 10.1002/jgrd.50613 (2013).

[47] Liu, H., Jacob, D. J., Bey, I. & Yantosca, R. M. Constraints from 210pb and 7be on wet deposition and transport in a global three-dimensional chemical tracer model driven by assimilated meteorological fields. *J. Geophys. Res. Atmospheres* **106**, 12109–12128, 10.1029/2000JD900839 (2001). https://agupubs.onlinelibrary.wiley.com/doi/pdf/10.1029/2000JD900839.

[48] Grandey, B. S., Gururaj, A., Stier, P. & Wagner, T. M. Rainfall-aerosol relationships explained by wet scavenging and humidity. *Geophys. Res. Lett.* **41**, 5678–5684 (2014).

[49] Gryspeerdt, E., Stier, P. & Partridge, D. Links between satellite-retrieved aerosol and precipitation. *Atmospheric Chem. Phys.* **14**, 9677–9694 (2014).

[50] Shingler, T. et al. Ambient observations of hygroscopic growth factor and f (rh) below 1: Case studies from surface and airborne measurements. *J. Geophys. Res. Atmospheres* **121**, 13–661 (2016). 10.1002/2016JD025471 PMC7821680 33489645

[51] Jiao, C. et al. An aerocom assessment of black carbon in arctic snow and sea ice. *Atmospheric Chem. Phys.* **14**, 2399–2417 (2014).

[52] Wu, M. et al. Impacts of aerosol dry deposition on black carbon spatial distributions and radiative effects in the community atmosphere model cam5. *J. Adv. Model. Earth Syst.* **10**, 1150–1171 (2018).

[53] Stohl, A. et al. Arctic smoke-record high air pollution levels in the european arctic due to agricultural fires in eastern europe in spring 2006. *Atmospheric Chem. Phys.* **7**, 511–534 (2007).

[54] Matsui, H. et al. Seasonal variation of the transport of black carbon aerosol from the asian continent to the arctic during the arctas aircraft campaign. *J. Geophys. Res. Atmospheres* **116** (2011).

[55] Willis, M. D. et al. Evidence for marine biogenic influence on summertime arctic aerosol. *Geophys. Res. Lett.* **44**, 6460–6470 (2017).

[56] Eckhardt, S. et al. The north atlantic oscillation controls air pollution transport to the arctic. *Atmospheric Chem. Phys.* **3**, 1769–1778 (2003).

[57] Myhre, G. et al. Radiative forcing of the direct aerosol effect from aerocom phase ii simulations. *Atmospheric Chem. Phys.* **13**, 1853–1877 (2013).

[58] Schmale, J. et al. Pan-arctic seasonal cycles and long-term trends of aerosol properties from 10 observatories. *Atmospheric Chem. Phys.* **22**, 3067–3096. 10.5194/acp-22-3067-2022 (2022).

[59] Gong, X. et al. Arctic warming by abundant fine sea salt aerosols from blowing snow. *Nat. Geosci.*10.1038/s41561-023-01254-8 (2023). 37692903 10.1038/s41561-023-01254-8 PMC10482690

[60] Baccarini, A. et al. Frequent new particle formation over the high arctic pack ice by enhanced iodine emissions. *Nat. communications* **11**, 1–11. 10.1038/s41467-020-18551-0 (2020). 10.1038/s41467-020-18551-0 PMC7529815 33004812

[61] Whaley, C. H. et al. Model evaluation of short-lived climate forcers for the arctic monitoring and assessment programme: a multi-species, multi-model study. *Atmospheric Chem. Phys.* **22**, 5775–5828 (2022).

[62] Raut, J.-C. et al. Cross-polar transport and scavenging of siberian aerosols containing black carbon during the 2012 access summer campaign. *Atmospheric Chem. Phys.* **17**, 10969–10995 (2017).

[63] Alexander, B. et al. Sulfate formation in sea-salt aerosols: Constraints from oxygen isotopes. *J. Geophys. Res. Atmospheres* **110**, 10.1029/2004JD005659 (2005). https://agupubs.onlinelibrary.wiley.com/doi/pdf/10.1029/2004JD005659.

[64] Fountoukis, C. & Nenes, A. Isorropia ii: a computationally efficient thermodynamic equilibrium model for k-ca-mg-nh-na-so-no-cl-ho aerosols. *Atmospheric Chem. Phys.* **7**, 4639–4659. 10.5194/acp-7-4639-2007 (2007).

[65] Knippertz, P. et al. The dacciwa project: Dynamics–aerosol–chemistry–cloud interactions in west africa. *Bull. Am. Meteorol. Soc.* **96**, 1451–1460. 10.1175/BAMS-D-14-00108.1 (2015).

[66] Eyring, V. et al. Overview of the coupled model intercomparison project phase 6 (cmip6) experimental design and organization. *Geosci. Model. Dev.* **9**, 1937–1958. 10.5194/gmd-9-1937-2016 (2016).

[67] Zhao, A., Ryder, C. L. & Wilcox, L. J. How well do the cmip6 models simulate dust aerosols?. *Atmospheric Chem. Phys.* **22**, 2095–2119. 10.5194/acp-22-2095-2022 (2022).

[68] Park, R. J., Jacob, D. J., Chin, M. & Martin, R. V. Sources of carbonaceous aerosols over the united states and implications for natural visibility. *J. Geophys. Res. Atmospheres* **108**, 10.1029/2002JD003190 (2003). https://agupubs.onlinelibrary.wiley.com/doi/pdf/10.1029/2002JD003190.

[69] Park, R. J. et al. Export efficiency of black carbon aerosol in continental outflow: Global implications. *J. Geophys. Res. Atmospheres* **110**, 10.1029/2004JD005432 (2005). https://agupubs.onlinelibrary.wiley.com/doi/pdf/10.1029/2004JD005432.

[70] Jaeglé, L., Quinn, P. K., Bates, T. S., Alexander, B. & Lin, J.-T. Global distribution of sea salt aerosols: new constraints from in situ and remote sensing observations. *Atmospheric Chem. Phys.* **11**, 3137–3157. 10.5194/acp-11-3137-2011 (2011).

[71] Koepke, P., Hess, M., Schult, I. & Shettle, E. P. Global aerosol data set. Tech. Rep. 243, Max-Planck-Institut fur Meteorologie (1997).

[72] Drury, E. et al. Synthesis of satellite (modis), aircraft (icartt), and surface (improve, epa-aqs, aeronet) aerosol observations over eastern north america to improve modis aerosol retrievals and constrain surface aerosol concentrations and sources. *J. Geophys. Res. Atmospheres* **115**, 10.1029/2009JD012629 (2010). https://agupubs.onlinelibrary.wiley.com/doi/pdf/10.1029/2009JD012629.

[73] Ridley, D. A., Heald, C. L., Pierce, J. R. & Evans, M. J. Toward resolution-independent dust emissions in global models: Impacts on the seasonal and spatial distribution of dust. *Geophys. Res. Lett.* **40**, 2873–2877, 10.1002/grl.50409 (2013). https://agupubs.onlinelibrary.wiley.com/doi/pdf/10.1002/grl.50409.

[74] Fisher, J. A. et al. Sources, distribution, and acidity of sulfate-ammonium aerosol in the arctic in winter-spring. *Atmospheric Environ.* **45**, 7301–7318. 10.1016/j.atmosenv.2011.08.030 (2011).

[75] model description and evaluation. Lu, X. et al. Development of the global atmospheric chemistry general circulation model bcc-geos-chem v1.0. *Geosci. Model. Dev.* **13**, 3817–3838. 10.5194/gmd-13-3817-2020 (2020).

[76] Hesaraki, S. et al. Comparisons of a chemical transport model with a four-year (april to september) analysis of fine-and coarse-mode aerosol optical depth retrievals over the canadian arctic. *Atmosphere-Ocean* **55**, 213–229 (2017).

[77] Martin, R. V., Jacob, D. J., Yantosca, R. M., Chin, M. & Ginoux, P. Global and regional decreases in tropospheric oxidants from photochemical effects of aerosols. *J. Geophys. Res. Atmospheres* **108**, 10.1029/2002JD002622 (2003). https://agupubs.onlinelibrary.wiley.com/doi/pdf/10.1029/2002JD002622.

[78] Mishchenko, M. I., Geogdzhayev, I. V., Cairns, B., Rossow, W. B. & Lacis, A. A. Aerosol retrievals over the ocean by use of channels 1 and 2 avhrr data: sensitivity analysis and preliminary results. *Appl. Opt.* **38**, 7325–7341. 10.1364/ao.38.007325 (1999). 18324281 10.1364/ao.38.007325

[79] Tegen, I. & Lacis, A. A. Modeling of particle size distribution and its influence on the radiative properties of mineral dust aerosol. *J. Geophys. Res. Atmospheres* **101**, 19237–19244, 10.1029/95JD03610 (1996). https://agupubs.onlinelibrary.wiley.com/doi/pdf/10.1029/95JD03610.

[80] Holben, B. N. et al. Aeronet—a federated instrument network and data archive for aerosol characterization. *Remote sensing of environment* **66**, 1–16. 10.1016/S0034-4257(98)00031-5 (1998).

[81] O’Neill, N. T., Eck, T. F., Smirnov, A., Holben, B. N. & Thulasiraman, S. Spectral discrimination of coarse and fine mode optical depth. *J. Geophys. Res. Atmospheres* **108**, 10.1029/2002JD002975 (2003). https://agupubs.onlinelibrary.wiley.com/doi/pdf/10.1029/2002JD002975.

[82] Saha, A. et al. Pan-arctic sunphotometry during the arctas-a campaign of april 2008. *Geophys. Res. Lett. ***37**, 10.1029/2009GL041375 (2010). https://agupubs.onlinelibrary.wiley.com/doi/pdf/10.1029/2009GL041375.

