# Supplementary material for: Model simulations capture seasonal Arctic Haze and clean-air cycle better than satellite and reanalysis
Source: Sci Rep. 2025 Dec 1;15:42934. doi: 10.1038/s41598-025-29188-8 (PMC12672705; doi:10.1038/s41598-025-29188-8)
Supplement: Supplementary file 1 — Supplementary Information. [file 41598_2025_29188_MOESM1_ESM.pdf]

Model simulations capture seasonal Arctic Haze and Clean Air cycle better than Satellite and Reanalysis:- Supplement

Basudev Swain[1,*], Marco Vountas[2], Aishwarya Singh[3], Rui Song[1], Upasana Panda[4], Heiko Schellhorn[2], Linus Andrae[2], Adrien Deroubaix[5], Luca Lelli[6], Ankit Tandon[7], Akshaya Nikumbh[8], and Sachin S. Gunthe[9]

[1,*]Department of Physics, Atmospheric, Oceanic and Planetary Physics, University of Oxford, UK
[2]Institute of Environmental Physics, University of Bremen, Germany
[3]Aerosol Chemistry Department, Max-Planck Institute for Chemistry, Mainz, Germany
[4]Kalinga Institute of Industrial Technology (KIIT) Deemed to be University, Bhubaneswar, India
[5]Max-Planck-Institut für Meteorologie, Hamburg, Germany
[6]Remote Sensing Technology Institute, German Aerospace Centre (DLR), Wessling, Germany
[7]Department of Environmental Sciences, Central University of Jammu, Jammu, India
[8]Department of Climate Studies, Indian Institute of Technology Bombay, Maharashtra, India
[9]Department of Civil Engineering, Indian Institute of Technology Madras, Chennai, India
[*]Corresponding, Basudev Swain: basudev.swain@physics.ox.ac.uk

1 Emission inventories used

The various emission inventories considered in this study is presented in Table 1 below.

**Emission Component**	**GEOS-Chem Inventory**	**CAMSRA Inventory**	**MERRA2 Inventory**	**Citation**
Aerosol Components (BC, OC)	CEDS	CEDS	CEDS	Hoesly et al. (2018)
Aerosol Precursor	CEDS	CEDS	CEDS	Hoesly et al. (2018)
SO_2, NOx, NH_3, CH_4, CO, NMVOC	CEDS	CEDS	CEDS	Hoesly et al. (2018)
CO_2	CEDS	CEDS	CEDS	Hoesly et al. (2018)
Aircraft Emissions	AEIC	AEIC	AEIC	Simone et al. (2013)
Biofuel and Agricultural Burning	Biofuel	GFED	GFED	Yevich and Logan (2003)
US & Mexico Inventory (BRAVO)	BRAVO	NEI	NEI	Kuhns et al. (2005)
Ammonia (NH_3) Canada	APEI	EMEP	EMEP	Sheppard et al. (2010)
European Emissions	EMEP	EMEP	EMEP	Auvray et al. (2007)
Natural, Biofuel NH_3	GEIA	GEIA	GEIA	Bouwman et al. (1997); Croft et al. (2016)
US EPA Emissions	EPA/NEI2011	NEI	NEI	Simon et al. (2010)
Anthropogenic VOCs	RETRO	RETRO	RETRO	Bolshcer et al. (2007)
Asian Emissions	MIX	MIX	MIX	Li et al. (2017)
African Emissions	DICE-Africa	DICE	DICE	Marais and Wiedinmyer (2016)
Biomass Burning Emissions	GFED4	GFED4	GFED4	Giglio et al. (2013)
Volcanic SO_2 Emissions	Volcanic Inventory	Volcanic Inventory	Volcanic Inventory	Fisher et al. (2011)
Sea Salt Aerosols	Sea Salt	Sea Salt	Sea Salt	Jaeglé et al. (2011)
Mineral Dust	Mineral Dust	Mineral Dust	Mineral Dust	Zender et al. (2003); Fairlie et al. (2007)
Shipping Emissions	CEDS SHIP, EMEP SHIP	CEDS SHIP, EMEP SHIP	CEDS SHIP, EMEP SHIP	Hoesly et al. (2018)
Stratospheric Volcanic Emissions	Kasatochi and Sarychev	Kasatochi, Sarychev	Kasatochi, Sarychev	Fisher et al. (2011); Carn et al. (2015)

Table S1. Emission inventories used in GEOS-Chem, CAMSRA, and MERRA2 in this study.

References

Auvray, M., Bey, I., Llull, E., Schultz, M. G., and Rast, S.: A model investigation of tropospheric ozone chemical tendencies in long-range transported pollution plumes, Journal of Geophysical Research: Atmospheres, 112, https://doi.org/10.1029/2006JD007137, 2007.

Bolshcer, M. et al.: RETRO Deliverable D1-6, RETRO documentation, 2007.

Bouwman, A. F., Lee, D. S., Asman, W. A. H., Dentener, F. J., Van Der Hoek, K. W., and Olivier, J. G. J.: A global high-resolution emission inventory for ammonia, Global Biogeochemical Cycles, 11, 561–587, https://doi.org/10.1029/97GB02266, 1997.

Carn, S. A., Yang, K., Prata, A. J., and Krotkov, N. A.: Extending the long-term record of volcanic SO2 emissions with the Ozone Mapping and Profiler Suite nadir mapper, Geophysical Research Letters, 42, 925–932, https://doi.org/10.1002/2014GL062437, 2015.

Croft, B., Martin, R. V., Leaitch, W. R., Tunved, P., Breider, T. J., D'Andrea, S. D., and Pierce, J. R.: Processes controlling the annual cycle of Arctic aerosol number and size distributions, Atmospheric Chemistry and Physics, 16, 3665–3682, https://doi.org/10.5194/acp-16-3665-2016, 2016.

Fairlie, T. D., Jacob, D. J., and Park, R. J.: The impact of transpacific transport of mineral dust in the United States, Atmospheric Environment, 41, 1251–1266, https://doi.org/10.1016/j.atmosenv.2006.09.048, 2007.

Fisher, J. A., Jacob, D. J., Wang, Q., Bahreini, R., Carouge, C. C., Cubison, M. J., Dibb, J. E., Diehl, T., Jimenez, J. L., Leibensperger, E. M., et al.: Sources, distribution, and acidity of sulfate–ammonium aerosol in the Arctic in winter–spring, Atmospheric Environment, 45, 7301–7318, https://doi.org/10.1016/j.atmosenv.2011.08.030, 2011.

Giglio, L., Randerson, J. T., and van der Werf, G. R.: Analysis of daily, monthly, and annual burned area using the fourth-generation global fire emissions database (GFED4), Journal of Geophysical Research: Biogeosciences, 118, 317–328, https://doi.org/10.1002/jgrg.20042, 2013.

Hoesly, R. M., Smith, S. J., Feng, L., Klimont, Z., Janssens-Maenhout, G., Pitkanen, T., Seibert, J. J., Vu, L., Andres, R. J., Bolt, R. M., Bond, T. C., Dawidowski, L., Kholod, N., Kurokawa, J.-I., Li, M., Liu, L., Lu, Z., Moura, M. C. P., O'Rourke, P. R., and Zhang, Q.: Historical (1750–2014) anthropogenic emissions of reactive gases and aerosols from the Community Emissions Data System (CEDS), Geoscientific Model Development, 11, 369–408, https://doi.org/10.5194/gmd-11-369-2018, 2018.

Jaeglé, L., Quinn, P. K., Bates, T. S., Alexander, B., and Lin, J.-T.: Global distribution of sea salt aerosols: new constraints from in situ and remote sensing observations, Atmospheric Chemistry and Physics, 11, 3137–3157, https://doi.org/10.5194/acp-11-3137-2011, 2011.

Kuhns, H., Knipping, E. M., and Vukovich, J. M.: Development of a United States–Mexico emissions inventory for the big bend regional aerosol and visibility observational (BRAVO) study, Journal of the Air & Waste Management Association, 55, 677–692, https://doi.org/10.1080/10473289.2005.10464648, 2005.

Li, M., Zhang, Q., Kurokawa, J.-I., Woo, J.-H., He, K., Lu, Z., Ohara, T., Song, Y., Streets, D. G., Carmichael, G. R., Cheng, Y., Hong, C., Huo, H., Jiang, X., Kang, S., Liu, F., Su, H., and Zheng, B.: MIX: a mosaic Asian anthropogenic emission inventory under the international collaboration framework of the MICS-Asia and HTAP, Atmospheric Chemistry and Physics, 17, 935–963, https://doi.org/10.5194/acp-17-935-2017, 2017.

Marais, E. A. and Wiedinmyer, C.: Air quality impact of diffuse and inefficient combustion emissions in Africa (DICE-Africa), Environmental science & technology, 50, 10 739–10 745, https://doi.org/10.1021/acs.est.6b02602, 2016.

Sheppard, S., Bittman, S., and Bruulsema, T.: Monthly ammonia emissions from fertilizers in 12 Canadian Ecoregions, Canadian Journal of Soil Science, 90, 113–127, https://doi.org/10.4141/CJSS09006, 2010.

Simon, H., Beck, L., Bhave, P. V., Divita, F., Hsu, Y., Luecken, D., Mobley, J. D., Pouliot, G. A., Reff, A., Sarwar, G., and Strum, M.: The development and uses of EPA's SPECIATE database, Atmospheric Pollution Research, 1, 196–206, https://doi.org/10.5094/APR.2010.026, 2010.

Simone, N., Stettler, M., Eastham, S., and Barrett, S.: An open global civil aviation emissions dataset for 2005 (R1), Tech. rep., MIT Laboratory for Aviation and the Environment, http://nehalem001.mit.edu/uploads/LAE_report_series/2013/LAE-2013-001-N.pdf, 2013.

Yevich, R. and Logan, J. A.: An assessment of biofuel use and burning of agricultural waste in the developing world, Global Biogeochemical Cycles, 17, https://doi.org/10.1029/2002GB001952, 2003.

Zender, C. S., Bian, H., and Newman, D.: Mineral Dust Entrainment and Deposition (DEAD) model: Description and 1990s dust climatology, Journal of Geophysical Research: Atmospheres, 108, https://doi.org/10.1029/2002JD002775, 2003.
